# Increasing Thermal Stability of Gelatin by UV-Induced Cross-Linking with Glucose

**DOI:** 10.1155/2014/979636

**Published:** 2014-05-21

**Authors:** Evan M. Masutani, Christopher K. Kinoshita, Travis T. Tanaka, Andrew K. D. Ellison, Brandon A. Yoza

**Affiliations:** ^1^Stanford University, Stanford, CA 94305, USA; ^2^Hawaii Natural Energy Institute, University of Hawaii, 1680 East West Road, POST 109, Honolulu, HI 96822, USA; ^3^University of Pennsylvania, Philadelphia, PA 19104, USA

## Abstract

The effects of ultraviolet (254 nm) radiation on a hydrated gelatin-glucose matrix were investigated for the development of a physiologically thermostable substrate for potential use in cell scaffold production. Experiments conducted with a differential scanning calorimeter indicate that ultraviolet irradiation of gelatin-glucose hydrogels dramatically increases thermal stability such that no melting is observed at temperatures of at least 90°C. The addition of glucose significantly increases the yield of cross-linked product, suggesting that glucose has a role in cross-link formation. Comparisons of lyophilized samples using scanning electron microscopy show that irradiated materials have visibly different densities.

## 1. Introduction

Pursuit of* in vitro* biomimetic organ growth has spurred a number of recent investigations of methods to generate three-dimensional (3D) scaffolding structures and techniques for cellular seeding [1–3].* In vitro* organs that have demonstrated functionality similar to natural organs have been produced previously by cell reseeding onto cadaver-derived, decellularized protein scaffolds [4]. These results suggest that the development of a sufficiently complex 3D cell scaffold may allow the regrowth of organs* de novo*.

Cell scaffolding structures, commonly referred to as extracellular matrices (ECM), should be constructed from benign compounds [5]. Scaffold materials will either decompose metabolically during cell propagation or be fully incorporated into the final organ. Materials that have been suggested include ceramics, chitosan, collagen, peptides, polyethylene glycol (PEG), polysaccharides, and various synthetic biomaterials [6]. For applications involving human hosts, material selection criteria must consider toxicity, antigenicity, mechanical strength, thermal stability, and porosity.

Collagen has been used frequently in previous investigations of ECM development [6–8]. It is a crystalline [9–11], triple helical molecule [12] and a favorable material for biomedical applications, since it is a biodegradable and biocompatible insoluble fibril with high mechanical strength and relatively low immunogenicity [13–15].

Gelatin is the incompletely denatured form of collagen and comprises variable-length peptides which have fibrillar structure but lack configurational order [16].* In vivo* use of gelatin has been successfully demonstrated by implantation in animal models, with results that suggest low toxicity and reduced antigenicity relative to collagen [17, 18]. Furthermore, gelatin is relatively inexpensive compared to collagen and its cell adhesion and proliferation characteristics are essentially indistinguishable [19]. Gelatin's use in ECM is complicated by its lack of 3D structural integrity, lower melting temperatures, and rapid dissolution in water [16, 20]. For use as cell scaffolds, recent studies have sought to increase the mechanical and thermal resiliency through compositing with various compounds [21, 22] and by utilization of covalent cross-linking agents [23]. Many cross-linking agents, however, are toxic or immunogenic, for example, glutaraldehyde [8].

One potential method of cross-linking is the application of UV radiation to generate covalent intermolecular bonds. Otoni et al. demonstrated that application of UV-B to gelatin films greatly increased gel strength and viscosity [24]. The melting characteristics of the irradiated gels, however, remained relatively unchanged and thus the conditions employed are insufficient to generate gels which are thermostable at physiological conditions [24]. To improve the thermal stability of gelatin hydrogels, this study employs a combination of glucose and shorter UV-C radiation to cross-link gelatin.

The utilization of sugars as a gelatin cross-linking agent has been previously investigated [25], and its usefulness* in vivo* without host toxicity has been successfully demonstrated. Cross-linking between gelatin and both nonreducing and reducing sugars can be observed without catalysis; however, due to weak ionic interactions, dissolution still occurs at physiological temperatures (lower than 37°C), albeit at a reduced rate [25]. The formation of covalent interactions is therefore necessary to produce a thermostable compound. The Maillard reaction pathway is a potential chemistry that can generate covalent bonds between reducing sugars and protein amine groups [26] and produces physical changes in gelatin and other protein matrices [27–29].

Glycation end products are the resultant glycosylated proteins generated by Maillard chemistry [30, 31]. Sugar cross-linking of gelatin molecules has been shown to increase stiffness and decrease solubility [31, 32]. The present paper reports that ultraviolet (UV-C) radiation can provide the necessary energetic input required to cross-link gelatin, with increased yield in the presence of glucose. This is the first investigation to quantitatively describe the observed increased thermal stability using calorimetry. The cross-linked gelatin product demonstrates good thermal stability and has the potential for future 3D cell scaffold application.

## 2. Materials and Methods

### 2.1. Gelatin Film Preparation

Gelatin films were prepared using commercial gelatin powder type A (Fisher Scientific CAS number 9000-70-8), glucose (dextrose anhydrous; Fisher Scientific, CAS number 50-99-7), and distilled deionized water. The films were prepared gravimetrically with a Mettler AB-104S microbalance using 5 cm diameter aluminum weighing pans having a blend ratio of 2 : 1 : 2 w/w/w gelatin, glucose, and water [33]. The gelatin and glucose powder were homogenized in the pans. Distilled and deionized water was heated to approximately 100°C and added to the weighed gelatin and glucose. The mixture was quickly blended using a metal spatula until the material was homogenous and viscous. Before solidifying, the mixture was spread thinly across the aluminum pan. Samples having no added sugar were prepared using a 2 : 1 w/w ratio of gelatin and water.

### 2.2. Ultraviolet Irradiation

Samples were irradiated with 27 J/cm^2^ (2700 uW for 2 hours and 46 minutes) in a Spectroline UV cross-linker Model XL-1000 equipped with 254 nm bulbs. After irradiation, films were placed in 50 mL of distilled and deionized water then incubated at 45°C for 24 hours. Gelatin that was not successfully cross-linked after UV exposure solubilized in the distilled and deionized water. The remaining temperature-stable material was collected by decanting and triple rinsing with distilled water. Samples were then resuspended in distilled water and stored at 4°C until analysis. Nonirradiated control samples were hydrated in 50 mL of distilled deionized water and incubated at room temperature for 24 hours prior to refrigeration.

### 2.3. Dry Mass Analysis

To test for the effects of glucose on yield of cross-linked product, gelatin films were cast with and without glucose. The glucose containing films were prepared by homogenizing type A gelatin, glucose, and distilled water in a 2 : 1 : 2 w/v/w ratio. The glucose-deficient films were prepared by dissolving gelatin in distilled water in a 1 : 1 w/v ratio. Both types of films were prepared simultaneously in triplicate by the addition of 5 mL of material to a six-well tissue culture plate (Costar Cat number 3506) having a circular well surface area of 9.62 cm^2^. The surface of the gels was flattened by first covering and placing the tissue culture plate in a 45°C incubator. All gels were irradiated at 254 nm and were subsequently removed and submerged in 50 mL of distilled water in 100 mL Erlenmeyer flasks and placed in a 45°C incubator for 4 hours. The remaining solid material was kept by decanting the liquid and rinsed 3 times with 20 mL of water. The dry mass was calculated using an analytical balance and preweighed aluminum pan at 45°C 12 hrs.

### 2.4. Calorimetry

A TA Instruments multicell differential scanning calorimeter (Model MC DSC) having a detection limit of 0.2 mW was utilized for determination of melting temperature profiles. Instrument calibration was performed using fused silica as a reference standard. Gelatin films were lightly dried on weighing paper to remove excess water and approximately 0.3 g of sample was measured gravimetrically and placed into 1 mL hastelloy ampoules for testing. The MC DSC has four thermal wells: one reference and three samples. Triplicate samples were allowed to equilibrate in the calorimeter for 30 minutes at 10°C. The temperature was then increased linearly at a rate of 0.5°C/min and heat flow recorded having a resolution of 10 seconds from 10°C to 90°C. The upper limit of 90°C was utilized to avoid complications with the measurement associated with the water phase change. The samples were then allowed to dry in the open ampoules for 24 hours at 45°C. For accurate heat flow determination, calculations were performed utilizing gravimetrically determined dry samples mass.

### 2.5. Scanning Electron Microscopy

Scanning electron microscopy (SEM) was utilized to detect any visible small-scale surface structural differences in the UV-irradiated and nonirradiated samples. Prepared hydrated samples were freeze dried in a Labconco 2.5 Freezone for 24 hours. Subsamples of approximately 25 mg were then placed on aluminum support stubs covered with carbon tape and sputter coated with gold/palladium in a Hummer 6.2 sputter coater. Visualization was performed using a Hitachi S-4800 field emission SEM.

### 2.6. UV-Visible Spectrophotometry

UV-visible spectrophotometry was employed to detect differences in the transmission spectra between UV-irradiated and nonirradiated samples. The sample ratio was modified to a 2 : 1 : 16 w/w/w ratio of gelatin, glucose, and water to promote even spreading on fused silica windows. 100 *μ*L of liquefied sample was evenly spread on a 1′′ fused silica window. The nonirradiated gel-coated window was then dried at 45°C for two hours. The nonirradiated sample's percent transmittance was then measured in a Perkin Elmer Lambda 2 spectrophotometer from 190 nm to 1100 nm. The sample was then UV-irradiated with 27 J/cm^2^ and its percent transmittance was measured. The same sample was then placed inverted in a 45°C water bath for an hour to remove the colored byproduct produced by the glycolytic reaction, dried in the incubator again, and remeasured.

### 2.7. Gel Patterning

100 micron-scale patterns were generated on the gels through selective irradiation with UV. Patterns were generated and printed onto gel films with 10% wt ascorbic acid solution using a modified HP Deskjet 1000. The ascorbic acid functioned both as a photomask [34] and as a potential free radical scavenger [35] to prevent UV-induced cross-linking. The films were then exposed to 27 J/cm^2^ of UV and were immersed in 90°C water for 5 minutes. The gels were then recovered and examined with an Olympus BX-43 fluorescence microscope.

## 3. Results

**Figure 1:**
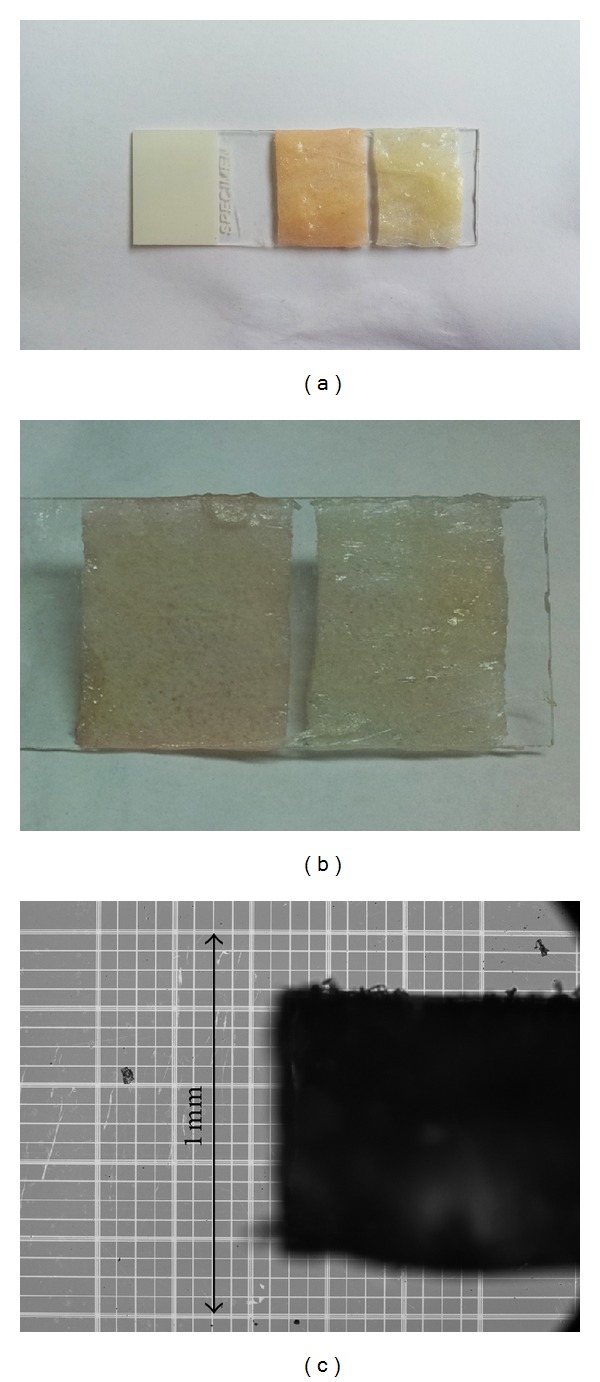
A single gelatin film was prepared using the described methodology and was placed on a glass slide, mechanically separated, and irradiated at 27 J/cm^2^. (a) UV-irradiated sample. (b) Control sample without irradiation (masked in aluminum foil). (c) Thickness of gel as seen by light microscopy.

Irradiation of gelatin-sugar samples results in the formation of a colored hydrogel as seen in the photograph presented in Figure 1(a). At the irradiation intensities employed in this investigation, this color change was confined to a thin layer at the material's surface. Figure 1(b) is a photograph of the back of the two samples shown in Figure 1(a). The difference in color between irradiated and nonirradiated samples is far less pronounced. The nominal cross sectional thickness of the films on the slide is about 0.75 mm (Figure 1(c)). Examination of the film cross sections with a light microscope (Olympus BX43) suggests that the color change penetrates less than 20% of the film thickness. Incubation of the irradiated material at 45°C in an aqueous solution dissolves the uncolored portion, leaving only a thin sheet of hydrated thermostable product. The observed color of the irradiated sample is mostly removed upon incubation in water. This suggests that the compound responsible for the color change is a byproduct of the cross-linking reaction and is not strongly associated with the newly cross-linked molecular arrangement. The exact compound requires further analysis.

Addition of glucose increased the yield of cross-linked product (Welch's* t*-test, *P* = 0.0181). The mean dry mass of UV-irradiated glucose containing films was 0.0889 g with a standard deviation of 0.0263 g, while the mean dry mass of UV-irradiated glucose-deficient films was 0.0122 g with a standard deviation of 0.0021 g. Comparison of the dry mass results for the glucose containing samples suggests that UV penetration and cross-linking occurs at a depth similar to what is visually seen by microscopy. Given the 40 percent water content in glucose containing gelatins the dry mass of the recovered material would correlate with a depth penetration that is approximately 0.15 mm.

In order to confirm and evaluate quantitatively the apparent thermostability of the UV-irradiated gelatin, samples were tested using differential scanning calorimetry (DSC) and compared to nonirradiated glucose containing controls. The gelatin polymers were hydrated using the protocol described in the preparation of ultraviolet treated samples prior to analysis with the MC DSC instrument in order to minimize errors associated with differences in water content. The actual dry weights of each sample, which were used to calculate heats of fusion from the DSC data, were determined postcalorimetry as described previously.

**Figure 2:**
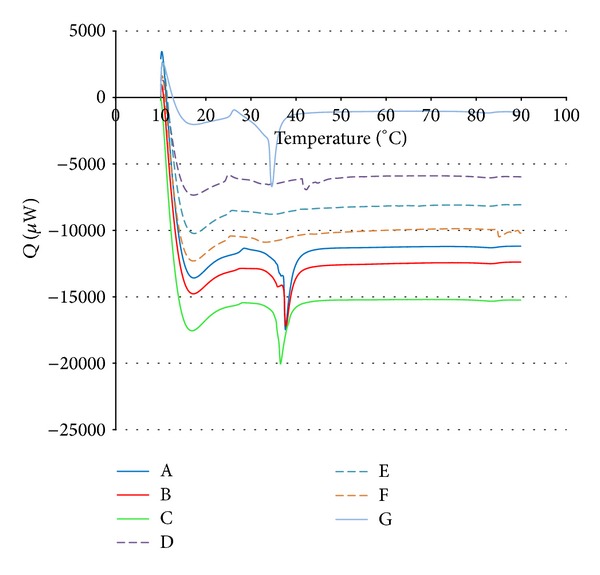
Calorimetric determination of gelatin samples prepared with and without UV irradiation. (A–C) Control samples with glucose and without irradiation. (D–F) UV-irradiated samples with glucose. (G) Control samples without glucose and without irradiation.

Figure 2 presents representative thermograms for triplicate replicates of the UV-irradiated gelatin-glucose samples and nonirradiated glucose containing controls. Additional replicates were analyzed with identical results. For comparison, Figure 2 also includes a single thermogram for a nonirradiated gelatin sample with no added glucose (Figure 2 G). The seven traces shown in Figure 2 are reported with slight vertical offsets for image clarity. The initial sharp descent in the curves reflects the transient state that occurs as heating begins following the 30-minute equilibration period at 10°C, during which the sample needed to be constantly cooled. Nonirradiated gelatin-glucose and gelatin (only) samples (Figure 2; A, B, C, and G) all exhibited a sudden, sharp increase in negative (endothermic) heat flow as temperature rose above 34°C. The average melting temperature of the nonirradiated gelatin-glucose controls was determined to be 34.60 ± 0.84°C from nine-replicate measurements. The average heat of fusion values were calculated using sample dry mass and by numeric integration of the chromatogram area. The nonirradiated samples had an average heat of fusion of 45.08 ± 2.35 J/g.

Thermograms of the irradiated samples (Figure 2; D, E, and F) exhibited no changes in heat flow over the measured temperature from 10°C to 90°C. Visual examination of the ampoule contents after calorimetric measurement confirms these results. The original conformations of the inserted irradiated samples are maintained, unlike nonirradiated controls that have melted and resolidified in the ampoule well.

**Figure 3:**
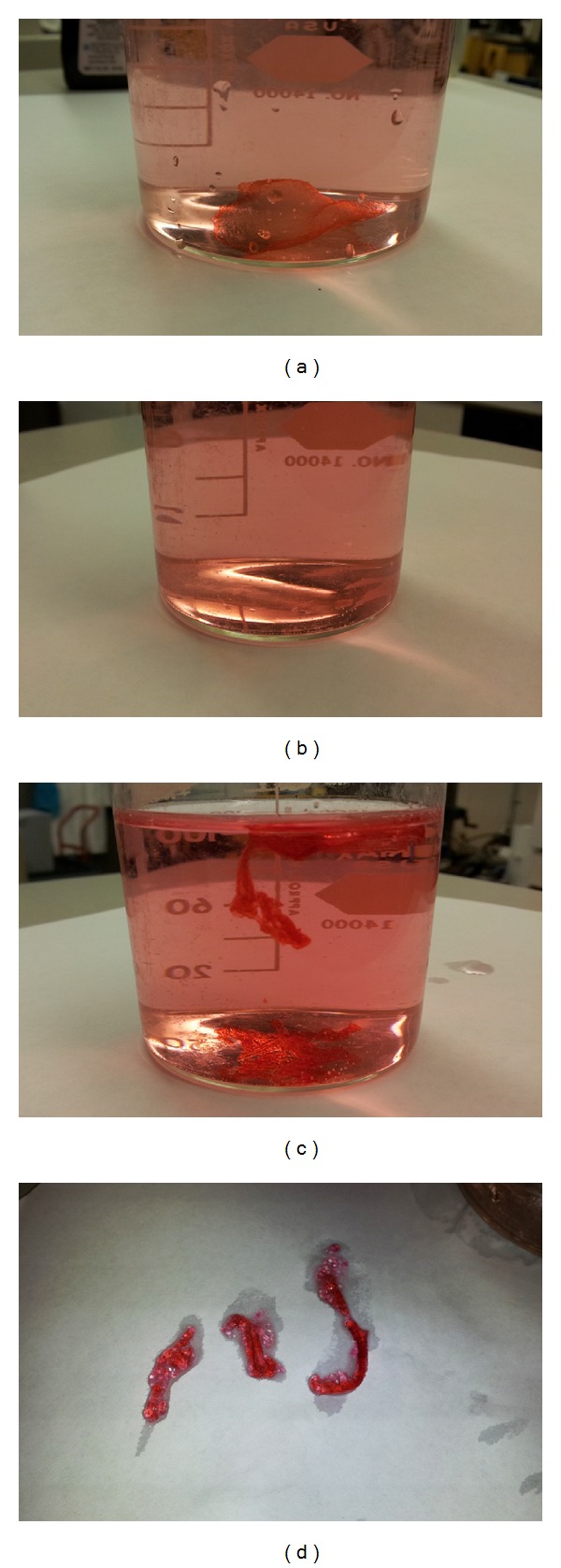
Irradiated samples were compared with samples without irradiation for temperature stability. These samples were prepared using the protocols described. Both samples were stained with red food dye and placed in an 85°C water bath. (a) Sample without irradiation before heating. (b) Sample without irradiation after heating. (c) Irradiated sample before heating. (d) Irradiated sample removed from water and shown after heating.

To visually illustrate the structural thermal stability of irradiated gelatin-glucose, samples were stained after hydration with a red food coloring dye and placed in an 85°C water bath for 30 minutes. As seen in Figures 3(a) and 3(b), the nonirradiated samples were no longer visible, having dissolved completely within 1 minute. The stable physical structure of the irradiated samples is shown in Figures 3(c) and 3(d) and did not change after 30 minutes of immersion.

**Figure 4:**
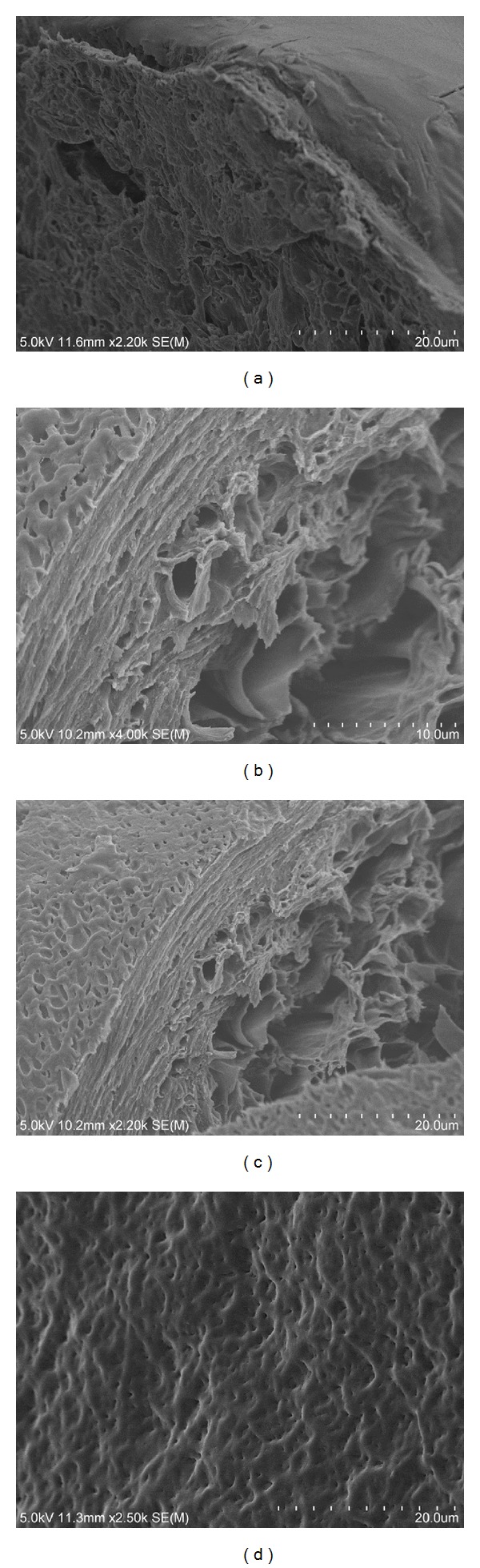
SEM comparisons of lyophilized irradiated samples and lyophilized nonirradiated samples. (a) Nonirradiated lyophilized sample surface and cross section. (b-c) Irradiated lyophilized sample cross sections at two different magnifications. (d) Irradiated lyophilized sample surface.

Nonirradiated and irradiated glucose containing samples were then lyophilized and observed with SEM. The nonirradiated sample's surface is very smooth and nonporous (Figure 4(a)) while the irradiated sample's surface appeared to be fibrillar and pockmarked (Figure 4(d)). Cross sectional images result in further visual differences. The irradiated sample appears to have a more stratified appearance, where increased density is observed at the surface interface which is directly exposed to ultraviolet irradiation (Figures 4(b) and 4(c)). The nonirradiated sample appears more homogenous with fewer surface features (Figure 4(a)). As previously mentioned, the surface penetration of the UV is limited to micron-scale depths. Cross-linking density may decrease with depth due to UV extinction.

**Figure 5:**
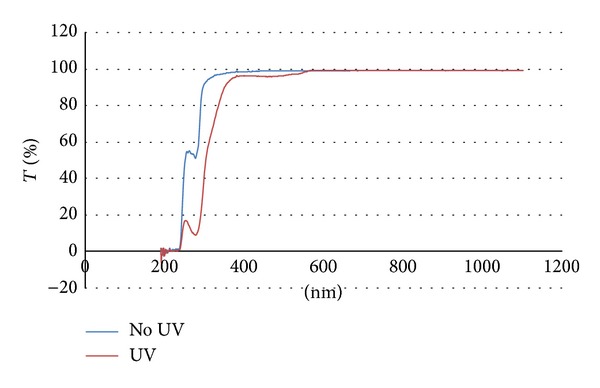
UV-Vis transmittance spectra for irradiated and nonirradiated gels. The red spectrum is that of the irradiated sample, while the blue spectrum is that of the nonirradiated sample.

Scanning UV-visible spectrophotometry was employed to detect absorbance changes in the gelatin composition after cross-linking. Figure 5 presents the percent transmittance of nonirradiated and UV-irradiated gelatin-glucose gels for wavelengths in the 190–1100 nm range. The peak centered at approximately 280 nm in the nonirradiated sample is potentially attributed to a convolution of absorbance by phenylalanine and tyrosine, which are present in small amounts [36]. Following UV irradiation, the peak centered at approximately 280 nm greatly increases in magnitude and another small peak appears at approximately 475 nm. The reductions in transmittance at 280 nm and at 475 nm were thought to be related to the cross-linking mechanism. Because some oxidation products of tyrosine absorb at around 475 nm, for example, dopachrome and aminochrome [37], it was posited that tyrosine oxidation was part of the potential cross-linking pathway. The peaks at 280 and 475 mm are still maintained after soaking irradiates samples in heated water.

**Figure 6:**
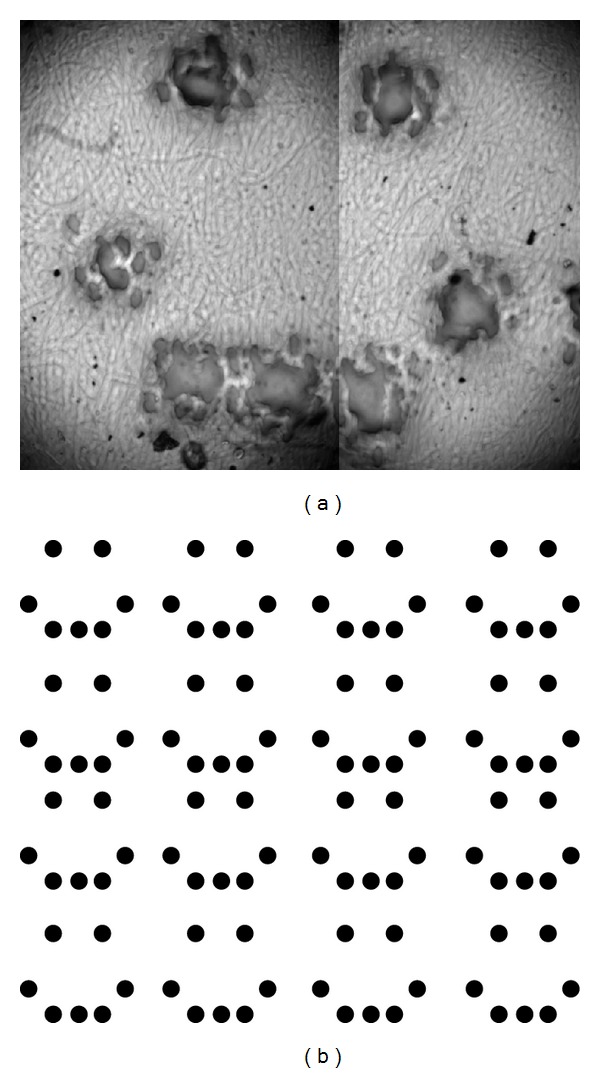
Selective irradiation of gels through application of ascorbic acid solution. (a) Resultant pattern after immersion in hot water under 40x magnification. (b) Original computer-generated pattern.

Lastly, Figure 6(a) depicts a sample of selectively UV-irradiated gel at 40x magnification, following immersion in 90°C water. Figure 6(b) depicts the original computer-generated pattern.

## 4. Discussion

Prior work has demonstrated that UV radiation-induced cross-linking increases the mechanical strength of collagen and collagen-sugar systems [30, 32, 38]. Gelatin is a denatured form of collagen and has similar chemical properties. Unlike collagen, gelatin is easily solubilized in liquid suspensions and can be cast into complex two- and three-dimensional shapes. Ionic interactions between the gelatin fibrils maintain a degree of structural integrity; however, gelatin rapidly dissolves in excess water and melts at physiological temperatures, which complicates its use as a material for biomimetic scaffolding structures.

Based on the similarities in the chemical structure of gelatin and collagen, and by analogy to the results of Ohan et al. [30], we posited that the application of UV radiation to a homogeneous gelatin-glucose substrate would promote cross-linking and, consequently, reduce solubility in aqueous media and enhance thermal stability. The results of our experimental investigation support this hypothesis. We were able to create a gelatin polymer that is stable at temperatures of at least 90°C.

There are multiple theories on the mechanism of cross-link formation in collagen. Due to the chemical similarity between collagen and gelatin, we expected that the same mechanisms would apply to gelatin. The first theory proposes that the collagen and sugar molecules undergo the Maillard reaction to form cross-links with neighboring collagen molecules [30]. One of the critical steps in the Maillard reaction is glycation, where the amine group of the protein attacks the reactive carbonyl group of the sugar [31]. Glycation requires the sugar to be in its linear chain form in order to have access to the reactive carbonyl group; typically, only 0.002% of glucose molecules are in the linear chain form [30]. It has been hypothesized that UV irradiation generates free radicals which react with the sugar molecules to increase the concentration of linear chain form sugar, which in turn increases the rate of glycation and cross-link formation [30].

Another mechanism is based on the observation that UV can promote cross-linking in collagen without sugar. Exposure to UV radiation at appropriate wavelengths generates free radicals on aromatic amino acids, for example, tyrosine and phenylalanine [38, 39], that can then form intermolecular bonds [38, 40].

We posit that UV irradiation generates free radicals in solution which accelerate cross-linking between individual gelatin molecules. Addition of antioxidant (L-ascorbic acid) inhibited the cross-linking process (Figure 6), agreeing with the results of Ohan et al. Based on our current results, it is difficult to ascertain the mechanism by which the free radicals react with the gelatin and sugar. Rather than one exclusive pathway for the formation of cross-links, multiple pathways can potentially exist and proceed in parallel. In the presence of sugar, gelatin undergoes the Maillard reaction and also forms bonds between its radical aromatic residues. Without sugar, gelatin only forms bonds between its radical aromatic residues. The results from the spectrophotometric experiments offer a potential mechanism for cross-link formation between aromatic residues.

The large decrease in transmittance following UV irradiation at 280 nm in Figure 5 is thought to be caused by either an increase in tyrosine content or the formation of dityrosyl groups. Typically, tyrosine, cysteine, tryptophan, and, to a much lesser extent, phenylalanine are assumed to be the primary contributors to absorbance at 280 nm [41]. Gelatin lacks tryptophan but has appreciable quantities of phenylalanine and a lesser amount of tyrosine [36]. It is unlikely for phenylalanine to form under these conditions; however, the conversion of phenylalanine into tyrosine as a result of UV irradiation with low efficiency has been documented [42]. Nevertheless, because the molar extinction coefficient of tyrosine at 280 nm in water is roughly an order of magnitude greater than that of phenylalanine in water, even partial conversion of phenylalanine to tyrosine would significantly reduce transmittance [43]. Even though the efficiency of phenylalanine conversion noted by Ishimitsu et al. is relatively low, their experiment used free amino acids whereas the current experimental setup has phenylalanine as part of a protein chain. The intermolecular interaction between gelatin chains and the intramolecular interactions between constituent amino acids may facilitate hydroxyl radical reactions with phenylalanine. Additionally, the absorbance peak that occurs at around 475 nm in the UV-irradiated samples may be indicative of dopachrome or aminochrome content, which are downstream products of tyrosine oxidation and cyclization [37, 42].

While the generation of tyrosine from phenylalanine will not result in cross-link formation, two tyrosyl radical groups can react to form a covalently-bound dityrosyl group. Ultraviolet radiation at 254 nm has been shown to generate tyrosine free radicals in aqueous solution [44]. The formation of dityrosyl cross-links could also contribute to the increased absorbance at 280 nm. The results of Rosei et al. indicate that dityrosine absorbs more strongly than tyrosine near 300 nm, with a primary peak at around 280 nm [45]. In Figure 5, the peak at 280 nm also appears to have broadened towards longer wavelengths, which could indicate dityrosyl formation. Experiments involving the UV irradiation of elastin have also proposed a similar mechanism for cross-linking [46].

The formation of dityrosyl groups is dependent on the presence of free radicals in solution [44]. The introduction of a free radical scavenger ascorbic acid as shown in Figure 6 strongly inhibits the formation of cross-links. From these data, the cross-linking mechanism is free radical dependent and the covalent cross-links are low in density. Dityrosyl bonds are durable and can withstand extreme conditions of prolonged incubation at 95°C in 6 N HCl [47]. Based on the reported durability of dityrosyl cross-links, such covalent bonds could be responsible for the increased thermal stability of UV-irradiated gelatin.

The addition of glucose to the reaction increased the yield of cross-linked product. It is possible that glucose increases cross-linking density, while not physically involved in polymer formation. Aqueous solutions of glucose and other sugars have been documented to degrade upon irradiation with wavelengths <300 nm releasing, peroxide species [47, 48]. Upon irradiation peroxide forms hydroxyl radicals [49] that oxidize phenylalanine forming tyrosine. Hydroxyl radicals can further oxidize tyrosine, resulting in radical production and the formation of a bityrosine covalent cross-link [50]. Further analysis is necessary to determine if this is one of the possible mechanistic pathways that results in increased thermostable polymer yield.

We were able to achieve reasonably high resolution in our selective irradiation of gel samples (Figures 6(a) and 6(b)). The smallest feature sizes were about 500 microns in diameter when swollen in water at 36°C. The feature sizes appeared to be limited by the resolution of the printhead, so it is likely that more specialized equipment could produce even finer details. Nevertheless, our smallest achievable pore sizes are comparable to some of the sizes required for tissue growth [51]. As opposed to conventional methods of pore formation, for example, electrospinning and freeze-drying, the position of every feature is planned beforehand in this method of selective irradiation. It could be possible to generate gelatin films with the same micron-scale geometry as native tissue scaffolds. Gelatin has many properties which make it an ideal starting material for cell scaffolds. Native gelatin is easily degraded by proteases, possesses minimal antigenicity [52, 53], and is very soluble in aqueous solution [54]. Based on our results, cross-linking gelatin renders it insoluble at physiological temperatures; prior work also correlates greater cross-linking density with reduced rate of enzymatic degradation [32]. By selectively irradiating sugar-gelatin mixtures with UV, we can generate regions with high resistance to dissolution and thermal degradation. Application of heat and/or protease will then remove the nonirradiated sectors of the gel, allowing the rapid and economical generation of complex gel geometries for use in cell scaffolds.

## 5. Conclusion

The current investigation has demonstrated the increased thermal stability and reduced water solubility of gelatin-sugar dispersions cross-linked by UV exposure. By increasing the melting temperature of gelatin, we have removed a major impediment for use of gelatin in tissue engineering applications. Since increased cross-link density generally correlates with increased mechanical strength and resistance to enzymatic degradation, it is also proposed that the described methodology enhances gelatin's ability to serve as a material for cell scaffold applications. The method of cross-link formation is posited to require the generation of free radicals and the formation of dityrosine between neighboring molecules. Future work will investigate whether a causal link between tyrosine content and cross-link formation exists. Through selective UV irradiation of gelatin-sugar dispersions, it should be possible to synthesize physiologically benign cell scaffolds with complex geometries for tissue engineering and possibly organ growth.
